# Interprofessional Discussion for Knowledge Transfer in a Digital “Community of Practice” for Managing Pneumoconiosis: Mixed Methods Study

**DOI:** 10.2196/67999

**Published:** 2025-03-13

**Authors:** Varinn Avi Sood, Heidi Rishel Brakey, Orrin Myers, Xin Shore, Akshay Sood

**Affiliations:** 1 Central New Mexico Community College, La Cueva High School, Albuquerque, NM, United States; 2 University of New Mexico Health Sciences Center, 1 University of New Mexico MSC 10 5550, Albuquerque, NM, 87131-0001, United States, 1 5052724751, 1 5052728700

**Keywords:** digital community of practice, knowledge transfer, pneumoconiosis, telementoring, rural health care, rural professionals, multidisciplinary management, interprofessional discussion, miner health, health equity, mixed methods, digital health, public health, digital community, self-efficacy, quantitative analyses, quantitative, technology, multidisciplinary care, patient outcome

## Abstract

**Background:**

Pneumoconiosis prevalence is increasing in the United States, especially among coal miners. Contemporaneously with an increased need for specialized multidisciplinary care for miners, there is a shortage of experts to fulfill this need. Miners’ Wellness ECHO (Extension for Community Health Outcomes) is a digital community of practice based on interprofessional discussion for knowledge transfer. The program has been demonstrated to increase participants’ self-efficacy for clinical, medicolegal, and “soft” skills related to miners’ health.

**Objective:**

We aimed to examine characteristics associated with interprofessional discussions and suggest ways to strengthen knowledge transfer.

**Methods:**

This mixed methods study used an exploratory sequential design. We video-recorded and transcribed ECHO sessions over 14 months from July 2018 to September 2019 and analyzed content to examine participant discussions. We focused on participants’ statements of expertise followed by other participants’ acceptance or eschewal of these statements (utterances). We conducted quantitative analyses to examine the associations of active participation in discussion (primary outcome variable, defined as any utterance). We analyzed the association of the outcome on the following predictors: (1) participant group status, (2) study time frame, (3) participant ECHO experience status, (4) concordance of participant group identity between presenter and participant, (5) video usage, and (6) attendance frequency. We used the generalized estimating equations approach for longitudinal data, logit link function for binary outcomes, and *LSMEANS* to examine least squares means of fixed effects.

**Results:**

We studied 23 sessions with 158 unique participants and 539 total participants, averaging 23.4 (SD 5.6) participants per session. Clinical providers, the largest participant group, constituting 36.7% (n=58) of unique participants, were the most vocal group (mean 21.74, SD 2.11 average utterances per person-session). Benefits counselors were the least vocal group, with an average utterance rate of 0.57 (SD 0.29) per person-session and constituting 8.2% (n=13) of unique participants. Thus, various participant groups exhibited different utterance rates across sessions (*P*=.003). Experienced participants may have dominated active participation in discussion compared to those with less or intermediate experience, but this difference was not statistically significant (*P*=.11). When the didactic presenter and participant were from the same participant group, active participation by the silent group participants was greater than when both were from different groups. This association was not seen in vocal group participants (interaction *P*=.003). Compared to those participating by audio, those participating on video tended to have higher rates of active participation, but this difference was not statistically significant (*P*=.11).

**Conclusions:**

Our findings provide insight into the mechanics of interprofessional discussion in a digital community of practice managing pneumoconiosis. Our results underscore the capacity of the novel ECHO model to leverage technology and workforce diversity to facilitate interprofessional discussions on the multidisciplinary care of miners. Future research will evaluate whether this translates into improved patient outcomes.

## Introduction

Mining dust-related diseases (eg, pneumoconiosis) are increasing in rural United States, especially among coal miners [1-4]. The 2017 prevalence of radiographic pneumoconiosis for coal miners with over 25 years of underground mining experience was greater than 10%, double the prevalence from the late 1990s [1]. Similarly, the 2014 rate of complicated pneumoconiosis (a particularly deadly form) among long-tenured underground coal miners was 1.1%, compared to 0.3% at its lowest point in the late 1990s [5].

Miners are medically vulnerable, underserved, and often underinsured. Many miners live in rural, remote, and mountainous locations in the Mountain West and Appalachia, constituting the “hot spot” regions of pneumoconiosis prevalence and mortality [6]. The prevalence of radiographic and complicated pneumoconiosis is the highest among all US miners in rural central Appalachia [7,8].

The re-emergence of pneumoconiosis presents unique challenges for rural communities. Compared with urban residents, those in rural areas have less access to outpatient pulmonary rehabilitation [9], primary care physicians [10], and pulmonologists [11]. Rural practitioners also face unique challenges, including professional isolation and complex patient profiles [12], and describe multiple barriers to knowledge acquisition, such as resources, personal costs, physical distance, and time [13].

Contemporaneously with an increase in the need for specialized multidisciplinary care for US miners, there is a shortage of experts to fulfill this need [14]. This critical gap is being addressed in New Mexico by innovative technology-based interventions such as mobile clinics equipped for telemedicine [15] and digital communities of practice for knowledge transfer. These interventions are recognized as rural and training innovations by the Rural Health Information Hub and the American Thoracic Society, respectively. The Miners’ Wellness ECHO (Extension for Community Health Outcomes) Program is a digital community of practice, defined as a group of people who “share a concern or a passion for something they do and learn how to do better as they interact regularly” [16].

Project ECHO began in 2003 to connect primary care providers in rural New Mexico at “spoke” sites to University-based experts at a “hub” site so the former could effectively treat their patients with hepatitis C locally, instead of referring them to the University of New Mexico [17]. Since then, the ECHO movement has grown exponentially, spanned many health topics, and reached people around the globe. The Miners’ Wellness ECHO Program provides structured longitudinal interprofessional telementoring to professionals caring for US miners in pneumoconiosis mortality hot spots, including respiratory therapists, home health professionals, benefits counselors, attorneys, clinical providers, and others.

Previous research of the Miners’ Wellness ECHO established that participants in the pneumoconiosis mortality hot spots in the United States valued telementoring sessions for delivering relevant, evidence-based, balanced, and objective content [18]. Participants also showed a significant increase in self-efficacy scores overall and for domains related to clinical, medicolegal, and “soft” skills pertaining to miners’ health, with “soft” skills including interpersonal and communication skills needed to navigate highly collaborative work in the care of miners [14]. The fact that existing participants showed greater improvement than fresh (newer) participants indicated that there might be a dose effect of telementoring on change in self-efficacy. In addition, greater improvement in outcomes among participants belonging to the clinical versus nonclinical professional groups might reflect the disproportionate emphasis on clinical didactics and case discussions in the program. Participants rated the collective efficacy of the virtual community of practice highly, stating feelings of being “closely knit,” trustworthiness, and willingness to help each other [19].

The foundation of knowledge transfer in the program is the “all teach, all learn” model, which implies that every participant has unique knowledge to contribute and share during interprofessional discussions. Our previous research showed that most Miners’ Wellness ECHO participants used knowledge ties from outside their participant group, emphasizing the interprofessional nature of knowledge transfer in miners’ care [20]. The knowledge transfer was more efficient for participants with greater ties to rural-based miners than those with lesser ties [20]. Given the compensation challenges faced by US miners, benefits counselors and attorneys played an outsized role in knowledge transfer [18]. The findings from the preliminary evaluation establish the usefulness of this unique program and provide the study rationale for further examination of its learning processes, communication, and participation, an area of limited scholarship [21].

The objective of our study was to examine characteristics associated with interprofessional discussions and suggest ways to strengthen knowledge transfer. The long-term goal of this study is to reduce health inequity through greater investment in interprofessional telementoring efforts that promote collaborative health care in medically underserved mining communities by fusing technology with specialized multidisciplinary expertise. This approach may help rural communities counter the re-emergence of the pneumoconiosis epidemic by ameliorating their shortage of skilled expertise in mining-related diseases.

## Methods

### ECHO Model

The ECHO approach differs from traditional telemedicine, where clinical providers (eg, physicians, physician assistants, and advanced practice providers) assume short-term direct care of individual patients. Further, unlike webinars or conventional didactic lectures, the ECHO model provides a digital discussion of cases with expert panels in real time that is highly contextualized and thereby fulfills key learning theories, such as deliberate practice [22], social cognitive theory [23], and situated learning and communities of practice [24]. As detailed in a previous publication [18], the ECHO model is based on the following key principles: (1) use of internet-based technology for multipoint videoconferencing, which helps leverage scarce resources; (2) use of an established evidence-based disease-management model that has been demonstrated to improve outcomes by reducing variation in processes of care and sharing best practices [17,25-27]; (3) use of case-based learning, based on discussion with experts and peers; (4) use of a digital community of practice, which emphasizes reciprocity in the sharing of skills and information; and (5) use of an internet-based database (ie, iECHO software [Hangzhou IECHO Science & Technology Co, Ltd]) to monitor outcomes.

### Miners’ Wellness ECHO Structure

Professionals involved in miners’ health are invited to attend the program using an emailed web link connected to a REDCap (Research Electronic Data Capture; Vanderbilt University) survey [28]. As published previously [18], the ECHO sessions are scheduled at the same time twice a month for 75 minutes, adhering to a standard format. Following a facilitator’s initial 10 minutes of introduction and announcements, an invited expert delivers a 15-minute didactic followed by a 20-minute facilitated question-answer session and a 30-minute digital facilitated case discussion. The medical director of the Miners’ Wellness ECHO program is trained to facilitate group discussions during a 3-day immersion program and is periodically retrained [29]. The ECHO format, employing adult learning principles, focuses more on active learning through discussion than on didactic training. Upon completing a session-specific survey, participants can receive continuing education credits without charge. A multidisciplinary committee of experts follows a structured curriculum that is continually adapted based on the needs of the virtual community of practice, which are identified through a review of the continuing evaluation reports.

Attendance at ECHO sessions is open and voluntary. Through session attendance, participants can participate in the didactic and case discussions, provide information and insight from their own experience, and receive mentoring from their peers and an expert panel. Outside the program sessions, participants continue to have access to peers and experts for urgent consultation requests via telephone or email. Over time, with iterative practice and feedback, participants gain additional expertise, and become more confident in their skill sets related to caring for miners in their respective fields [14,18,20]. Recorded didactic sessions are made available through a web-based archive. The didactic and case discussion topics were not repeated during this study’s time frame. All cases presented during this study’s time frame were resolved using the collective expertise of the multidisciplinary community of practice, which included experts. The University of New Mexico has a sustainable business model for its ECHO Programs, with the cost of personnel shared between university budgets and grants and contracts.

### Study Design and Recruitment

This 14-month exploratory sequential mixed methods study involved participants in the Miners’ Wellness ECHO Program from July 2018 to September 2019. Participants are invited from across the nation by email, flyers, and word of mouth referrals to join the ECHO sessions. Their session participation is voluntary. At the start of the session, participants are informed that the session is video recorded. Together, the University of New Mexico School of Medicine and its rural partner Miners’ Colfax Medical Center constitute the hub site of experts. The spoke partner sites are located across the pneumoconiosis mortality hot spot regions of the United States [18]. Although participants were allowed to attend multiple sessions, our analyses emphasize cross-sectional comparisons based on participant characteristics.

We asked the following 5 research questions on common ECHO processes based on the experience of this study’s team, which are also related to the engagement theory model that focuses on participation, engagement, and learning within communities of practice [30]:

1. Do some participant groups make more utterances than others (eg, do clinical providers make more utterances than benefit counselors)?
2. Do participants who are more experienced with ECHO have greater rates of active participation in discussion than those with lower levels of experience?
3. Does the participant group identity of the didactic presenter predict active participation in discussion from the same participant group during that session (eg, if a didactic presenter is an attorney, are other attorneys more likely to participate in discussion in that session)?
4. Does video usage predict active participation in discussion, as compared to audio usage?
5. Does session attendance frequency predict active participation in discussion?

### Video Recording and Transcription

Each ECHO session during this study time frame was video recorded and professionally transcribed. Participants were informed of the recording at the start of each session.

### Summative Qualitative Content Analysis

Using an exploratory approach from a naturalistic point of view, we conducted summative qualitative content analysis to code interprofessional discussions among ECHO participants in each video. Specifically, we examined participants’ verbal utterances, focusing on statements of expertise followed by acceptance or eschewal of the statements by other participants, using latent analysis for interpretation of content. We did not use a guiding theory, as this was exploratory. See Table 1 for the qualitative codebook with definitions of each. See Multimedia Appendix 1 for examples of conversations demonstrating statements of expertise and responses of acceptance and eschewal.

**Table 1.** Qualitative and quantitative content analysis codebook, from the mixed methods study of Miners’ Wellness ECHO^a^ participants, July 2018 to September 2019.

Variable, variable label, and variable code value				Definition or example of code value	Level of measurement
**Qualitative codebook**					
	**Utterance type^b^**				
		**What type of utterance did the participant make?**			
			1=Statement of expertise	Defining solutions to problems or answering questions. Anyone who speaks as an expert is an expert.	Nominal
			2=Acceptance of a statement of expertise	Others’ acceptance of a statement of expertise. Includes adding to statement (expanding is acceptance) or verbalizations such as “That’s great!” “Thank you so much for that.” “That’s really helpful!”	Nominal
			3=Eschewal of a statement of expertise	Others’ eschewal in response to a statement of expertise. Eschewal can include providing new “expertise” that contradicts statements in disagreement with previously stated opinions, information, evidence, or statements. Can include partial disagreement or eschewal.	Nominal
			4=Neutral or no response to a statement of expertise	Neutral or no response. For example, if a speaker moves to next question or topic.	Nominal
**Quantitative codebook**					
	**Number of utterances**				
		How many utterances did the participant make during the session?		Total number of expertise, acceptance, eschewal, or neutral statements during a session.	Ordinal
	**Participation**				
		**Did this person participate in a session?**			
			0=No	At least 1 utterance during a session	Nominal
			1=Yes	At least 1 utterance during a session	Nominal
	**Attendance**				
		**Did this person attend a session?**			
			0=No	Logged into a session	Nominal
			1=Yes	Logged into a session	Nominal
	**Session**				
		What is the session number?		23 sessions from July 2018 to September 2019	Ordinal
	**Video**				
		**Did the participant use their video camera during the session?**			
			1=Audio only	Use of video camera during a session	Nominal
			2=Video	Use of video camera during a session	Nominal
			3=Unknown	Use of video camera during a session	Nominal
	**Presenter**				
		**Was the participant a didactic presenter during the session?**			
			0=No	—^c^	Nominal
			1=Yes	—	Nominal
	**Participant group**				
		**What participant group was the person a part of?**			
			1=Attorney	Self-defined participant group status	Nominal
			2=Clinical provider	Self-defined participant group status	Nominal
			3=Home health professional	Self-defined participant group status	Nominal
			4=Respiratory therapist	Self-defined participant group status	Nominal
			5=Benefits counselor	Self-defined participant group status	Nominal
			6=Other	Self-defined participant group status	Nominal
	**Participant ECHO experience status**				
		**What joining group was the person a part of?**			
			1=Experienced	Participant joined before the start of this study, May 9, 2018.	Nominal
			2=Intermediate	Participant joined at the start of this study, May 9, 2018.	Nominal
			3=Less experienced	Joined after September 12, 2018.	Nominal

a ECHO: Extension for Community Health Outcomes.

b Utterances include statements of expertise, acceptance, and eschewal. Additional details are provided in Tables S1 and S2 in Multimedia Appendix 1.

c Not applicable.

We realize that evaluating others’ acceptance or eschewal can be subjective and difficult to determine by reading transcripts. Additionally, body language and tone of voice can convey meaning that written words cannot. To minimize these risks, our qualitative team consisted of 4 people with varied and complementary areas of expertise: (1) the principal investigator (AS) who is an expert pulmonologist and medical director/session facilitator for the Miners’ Wellness Tele-ECHO Clinic; (2) an expert qualitative methodologist (HRB); and (3) two experts of ECHO methods and processes (director of program operations, Rachelle Rochelle, MPA, and senior program manager, Stephen Murillo, MBA). This team of 4 analysts met frequently during the project to interpret session content and discuss coding, potential biases, and reflexivity. We concurrently coded video recordings of participant behavior alongside transcripts using NVivo (version 12; QSR International). The senior analyst created the preliminary codebook and independently coded the presence of each code across all ECHO sessions. She met with the principal investigator and 2 other team members to review the trial coding of the first 2 sessions as a group, modifying coding and definitions as needed. The principal investigator independently reviewed 2 additional sessions, and the other analysts independently reviewed 5 sessions; therefore, 7 sessions were trial-coded by 3 (5 sessions) or 4 (2 sessions) team members. The team regularly met to discuss coding until they were 100% in agreement, at which point the senior analyst coded the remaining 16 sessions. She continued to meet with the team periodically to discuss her coding generally and anything that was coded to be “unclear” (as defined in Table 1), at which point they jointly decided on whether it was better suited for another code. We removed all statements of expertise, acceptance, and eschewal of the session facilitator (AS), so we only evaluated participants’ utterances. This approach minimized the bias resulting from the effect of the role and relationships of the session facilitator (AS) in the data analysis. There were no unintended consequences from the program participation or analysis. Since thematic saturation was not the goal of this study, the sampling was guided by the number of sessions rather than sampling saturation.

### Quantitative Data Analysis

We worked with our senior statistician (OM) to convert our qualitative coding from NVivo into quantitative coding in Excel (Microsoft Corp). We coded additional variables to be analyzed with SAS (version 9.4; SAS Institute Inc). See Table 1 for the quantitative codebook.

### Outcome and Predictor Variables

We examined the primary outcome variable, “active participation in discussion,” defined by making any utterances (which included statements of expertise, acceptance, and eschewals). We analyzed the association of the outcome on the following predictor variables: (1) participant group status, (2) study time frame, (3) participant ECHO experience status, (4) concordance of participant group identity between presenter and participant, (5) video usage, and (6) attendance frequency.

Participant groups included attorneys, benefits counselors, clinical providers, home health professionals, respiratory therapists, and others. The participants’ ECHO experience status was determined by the participant’s date of joining the ECHO Program. Participants’ ECHO experience status was defined by (1) the experienced group: those who joined the Miners’ Wellness ECHO before the start of this study, May 9, 2018; (2) the intermediate group: those who joined at the start of this study, May 9, 2018; and (3) the less experienced group: those who joined after September 12, 2018. The participant groups were also classified as “silent” and “vocal” based on their preliminary analysis of their rates of utterances during the ECHO sessions. Participant group identity was considered concordant when the didactic presenter and participant belonged to the same participant group. Video status was determined if the video camera was turned on for any duration during a session by the participant.

### Analytic Strategy

The number of utterances by type (statements of expertise, acceptance, and eschewal) and overall for each participant and ECHO session were collected. An average number of utterances by participant group, session, and type were computed. Binary variables for any active participation in discussion (1=yes for ≥1 utterance in the session, 0=no for 0 utterances in the session) were made for each utterance type and overall. Frequencies and percentages for active participation variables and other categorical variables were compiled by utterance type, participant group, and session.

We used the generalized estimating equations approach to extend the generalized linear model to handle longitudinal data, including predictor variables. The logit link function was applied for binary outcomes. Repeated statements with the subject option identified each session as a cluster. *LSMEANS* were used to compute and compare least squares means of fixed effects. A 2-tailed *P* value less than .05 was considered statistically significant.

### Ethical Considerations

This study was an arm of a larger study. This arm of this study included participants who consented and enrolled in the larger study and those who were not enrolled in the larger study. This arm was approved as exempt by the institutional review board, University of New Mexico Human Research Protections Office (HRRC#18‐386). Informed consent was waived by the institutional review board for this arm, separate from the larger study. The study data were deidentified for analysis. No compensation was offered for this arm, separate from the larger study.

## Results

### Descriptive Characteristics

We studied 23 ECHO sessions during this study’s time frame, with 158 unique participants and 539 total participants. Further, 78 (49.4%) participants attended a single session, 28 (17.7%) attended 2 sessions, 11 (7%) attended 3 sessions, 13 (8.2%) attended 4‐5 sessions, 13 (8.2%) attended 6‐10 sessions, and 15 (9.5%) attended 11‐20 sessions (overall mean 3.4, SD 4.1 sessions). Individual session attendance averaged 23.4 (SD 5.6; median 24, IQR 8; range=13‐36) participants per session. During these sessions, 23 participants presented 23 patient cases, and 23 invited experts presented 23 didactics.

### Do Some Participant Groups Make More Utterances Than Others?

Clinical providers, the largest participant group constituting 36.7% (58/158) of unique participants, were also the most vocal group (mean 21.74, SD 2.11, average utterances per person-session; Table 2). Attorneys, while the smallest group in number (11/158, 6.9%), were the second most vocal group (mean 6.73, SD 4.29, average utterances per person-session). Benefits counselors and home health professionals were the least vocal groups, with average utterance rates of 0.57 (SD 0.29) and 2.86 (SD 2.24) per person-session, respectively, constituting 8.2% (13/158) and 22.8% (36/158) of unique participants, respectively. Thus, the various participant groups exhibited significantly different rates of utterances across sessions (*P*=.003).

**Table 2.** Summary of participant groups and their participation in discussion, with utterance rates per person-session across 23 Miners’ Wellness ECHO^a^ sessions during this study’s time frame from July 2018 to September 2019.^b^^,^^c^

Participant group	Total PAS^d^, n	Total PRAS^e^, n/N (%)	Unique PAS, n	Unique PRAS, n/N (%)	Total statements of expertise, n	Average expertise per person-session	Total statements of acceptance, n	Average acceptance per person-session	Total statements of eschewal, n	Average eschew per person-session	Total utterances across sessions, n	Average utterances per person-session
Attorney	54	54/539 (10)	11	11/158 (6.9)	77	3.5	58	2.64	13	0.59	148	6.73
Benefits counselor	87	87/539 (16.1)	13	13/158 (8.2)	6	0.26	7	0.3	0	0	13	0.57
Clinical provider	186	186/539 (34.5)	58	58/158 (36.7)	280	12.17	182	7.91	38	1.65	500	21.74
Home health professional	89	89/539 (16.5)	36	36/158 (22.8)	36	1.71	21	1	3	0.14	60	2.86
Other or unknown	63	63/539 (11.7)	27	27/158 (17.1)	56	2.43	48	2.09	7	0.3	111	4.83
Respiratory therapist	60	60/539 (11.1)	13	13/158 (8.2)	53	2.3	31	1.35	12	0.52	96	4.17
Total	539	539/539 (100)	158	158/158 (100)	508	22.09	347	15.09	73	3.17	928	40.35

a ECHO: Extension for Community Health Outcome.

b Utterances include statements of expertise, acceptance, and eschewal. Additional detail of utterances is provided in Table S1 in Multimedia Appendix 1.

c Details of individual ECHO session content are shown in Table S2 in Multimedia Appendix 1 to provide more context to data shown in Figure 1.

d PAS: participants across sessions.

e PRAS: participant representation in all sessions.

Some topics generated a lot of discussion (ie, high rates of utterances), while others did not (Figure 1). Overall, utterances per person-session did not change over time (*P*=.50, Figure 1). It should be noted that in session 17, there were no utterances—this is the only session facilitated by a different person who was an experienced ECHO participant and expert clinical provider but not a trained facilitator. As shown in Table 2, most utterances are statements of expertise, and most statements are accepted. As shown in Figure 1, some topics generate a lot of controversy during discussion (ie, high rates of eschewal), while others do not.

**Figure 1.**
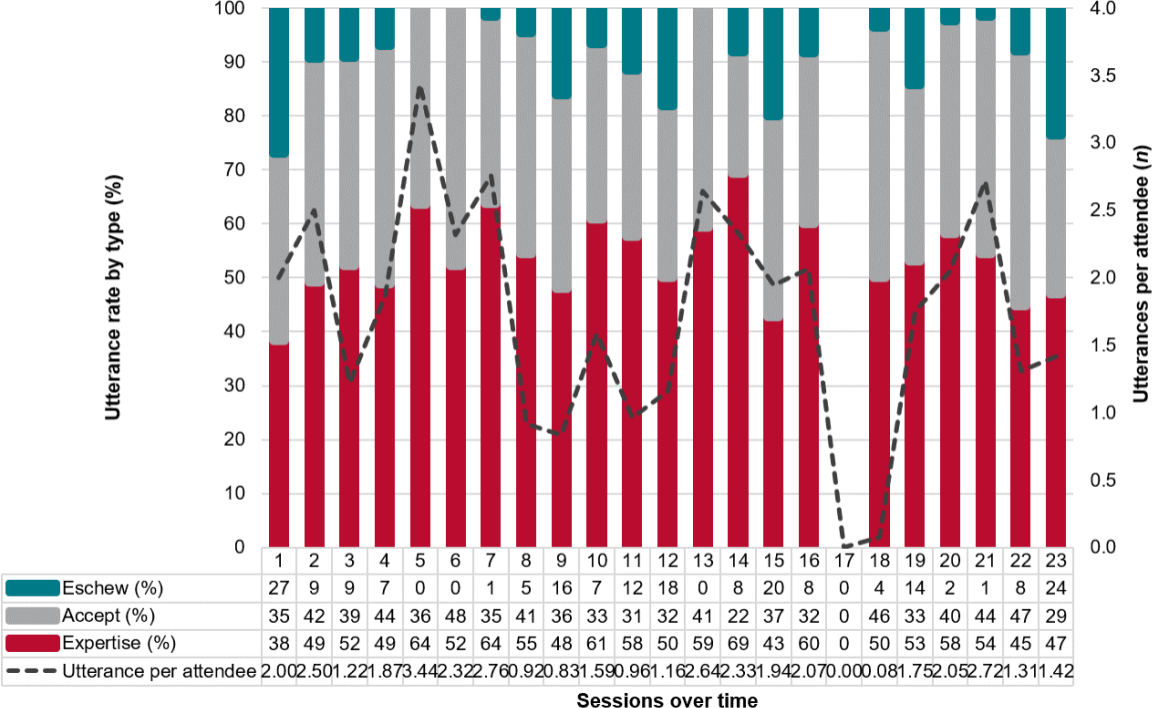
Rates of utterances across the 23 Miners’ Wellness ECHO sessions during this study‘s time frame, July 2018 to September 2019. This figure shows the average number of utterances per person-session (in gray line) and the percentage breakdown of each type of utterance (teal for eschewal, gray for acceptance, and red for statements of expertise). ECHO: Extension for Community Health Outcomes.

### Do Participants Who Are More Experienced With ECHO Have Greater Rates of Active Participation in Discussion Than Those With Lower Levels of Experience?

Experienced and intermediate experienced groups accounted for 36.8% (n=104) and 26.9% (n=29) of active participation in discussion, respectively, while the active participation from the less experienced group accounted for 28.6% (n=40). Experienced participants may have, thus, tended to dominate active participation in discussion compared to participants with less or intermediate levels of experience, but this difference was not statistically significant (*P*=.11, Table 3).

**Table 3.** Association of participants’ ECHO^a^ experience with active participation in discussion, as measured by any utterance during an individual session, during this study’s time frame from July 2018 to September 2019.

Group	Nonactive (n)	Active, n (%)	OR^b^ (95% CI)	*P* value	Overall *P* value
Experienced	179	104 (36.8)	1.45 (0.88-2.39)	.14	.11
Intermediate experience	79	29 (26.9)	0.92 (0.52-1.64)	.77	Referent
Less experienced	100	40 (28.6)	Referent	—^c^	Referent

a ECHO: Extension for Community Health Outcomes.

b OR: odds ratio.

c Not applicable.

### Does the Participant Group Identity of the Didactic Presenter Predict Active Participation in Discussion From the Same Participant Group During That Session?

Participant group identity was considered concordant when the didactic presenter and participant belonged to the same participant group. For this analysis, the participant groups were categorized as silent versus vocal, in which the benefits counselors and home health professionals constituted the former group and the clinical providers, attorneys and respiratory therapists in the latter group (with the other or unknown group excluded). When the didactic presenter and participant were from the same participant group, active participation in discussion (measured by any utterances within an individual ECHO session) by the silent group participants was 26.9% (n=7) compared to 9.5% (n=14) when the didactic presenter was from a different group (odds ratio 3.5, 95% CI 1.47-8.54, Table 4). Among vocal group participants, active participation was 46.4% (n=96) when presenters were in the same group as the participant, compared to 46.2% (n=43) when presenters and participants were from different groups (odds ratio 1.01, 95% CI 0.58-1.75), with an interaction *P*=.003.

**Table 4.** Association of active participation in Miners’ Wellness ECHO^a^ session discussion by silent and vocal groups on concordance of participant group identity between didactic presenter and participant, during this study’s time frame from July 2018 to September 2019.

Participant group concordance between presenter or participant^b^	Vocal group (n=300)			Silent group (n=173)			Interaction *P* value
	Nonactive (n)	Active, n (%)	OR^c^ (95% CI)	Nonactive (n)	Active, n (%)	OR (95% CI)	
Concordant	111	96 (46.4)	1.01 (0.58-1.75)	19	7 (26.9)	3.5 (1.47-8.54)	.003
Discordant	50	43 (46.2)	Referent	133	14 (9.5)	Referent	Referent

a ECHO: Extension for Community Health Outcomes.

b For this analysis, the participant groups were categorized as silent versus vocal, in which the benefits counselors and home health professionals constituted the former group and the clinical providers, attorneys and respiratory therapists in the latter group (with the other or unknown group excluded).

c OR: odds ratio.

### Does Video Usage Predict Active Participation in Discussion, as Compared to Audio Usage?

Compared to those joining a session by audio, those joining by video tended to have higher rates of active participation in discussion, as measured by any utterance during an individual session. However, this comparison did not reach statistical significance (Table 5, *P*=.11).

**Table 5.** Association of active participation in Miners’ Wellness ECHO^a^ session discussion on video camera use, during this study’s time frame from July 2018 to September 2019.

Group	Nonactive participation (n)	Active participation, n (%)	OR^b^ (95% CI)	*P* value
Audio^c^	110	54 (32.9)	Referent	.11
Video^c^	175	119 (40.5)	1.39 (0.93-2.07)	Referent

a ECHO: Extension for Community Health Outcomes.

b OR: odds ratio.

c Those whose audio and video status could not be determined (n=81) were excluded from the analysis.

### Does Session Attendance Frequency Predict Active Participation in Discussion?

Unique participants’ attendance ranged from 1 to 20 sessions and was categorized into 3 groups (Table 6). Overall, active participation in the discussion was significantly associated with attendance (*P*=.004); however, no dose response was noted.

**Table 6.** Association of active participation in Miners Wellness ECHO^a^ session discussion on frequency of session attendance, during this study’s time frame from July 2018 to September 2019.

Session attendance frequency	Nonactive participation (n)	Active participation, n (%)	OR^b^ (95% CI)	*P* value	Overall *P* value
≤5 sessions	76	56 (42.4)	Referent	—^c^	.004
6‐10 sessions	1	12 (92.3)	16.3 (2.1-128.9)	.008	Referent
11‐20 sessions	4	11 (73.3)	3.7 (1.1-12.3)	.03	Referent

a ECHO: Extension for Community Health Outcomes.

b OR: odds ratio.

c Not applicable.

## Discussion

### Principal Findings

In this digital community of practice for managing pneumoconiosis with previously published efficacy outcomes [14,18,20], our study findings show that some participant groups are vocal while others are silent, based on utterance rates during interprofessional discussion. Some topics generate a lot of discussion (ie, relatively high rates of utterances), and some topics generate a lot of controversy during discussion (ie, relatively high rates of eschewal of statements of expertise). Inadequately trained facilitators may impede discussion during a session. Experienced participants may dominate the discussion and inhibit participation by less experienced participants, but this association did not reach statistical significance (*P*=.11, Table 3). When the didactic presenter and participant are from the same participant group, participation in discussion by the silent group participants rises significantly. Compared to those participating by audio, those participating by video camera may tend to have higher rates of participation in discussion, but this association did not reach statistical significance. Overall, active participation in the discussion was significantly associated with attendance frequency. Our study findings lend themselves to several best practice recommendations, as discussed below.

The primary objective of the Project ECHO movement is to decentralize knowledge for the care of patients through exchanging insights and information using the all teach, all learn principle. All participants have unique knowledge sets, and discussion inside and outside the ECHO session within and across participant groups facilitates the transfer of knowledge that would otherwise remain siloed within individual participant groups. Further, interprofessional discussion in the ECHO model may allow greater access to new and thought-provoking ideas and perspectives that foster learning and other growth-enhancing actions. Our study findings confirm the existence of silent participant groups that, by not fully engaging in discussions, may not be as effective in exchanging knowledge (including providing and acquiring knowledge) within the ECHO session. These groups need to be actively supported by a trained facilitator and by the judicious use of didactic presenters sharing participant group identity concordant with the silent group participants. However, our previous objective measurement of knowledge transfer using social network principles indicates that benefits counselors are among the groups most effective in knowledge transfer [18]. This prior finding would suggest that either benefits counselors convey relevant knowledge succinctly within the ECHO sessions or participate in knowledge transfer outside the strict bounds of the ECHO program via follow-up phone calls, emails, and virtual and physical meetings. This finding warrants further research as it is a possible indication that the informal “social network” system, perhaps provoked by or aided by the ECHO sessions, may be more important than and reinforce the formal sessions.

Project ECHO formally trains its facilitators on the best practices for managing discussions in a digital community of practice. Substituting a trained facilitator by another untrained expert can affect participation in the discussion. Experienced participants usually offer the greatest rates of statements of expertise, acceptance, and eschewal, reflecting their expert status in this complex field. However, the dominance of experienced groups can be avoided by the facilitator actively encouraging less experienced groups to make their minority opinions heard during discussion. Using video cameras during participation in this digital community of practice helps build community and promotes accountability and engagement at the cost of rural internet bandwidth. This practice should be encouraged, even though the findings did not reach statistical significance.

Our study has multiple strengths. To the best of our knowledge, our approach of studying interprofessional discussion in a digital “community of practice” for managing pneumoconiosis has never been used previously by another group of investigators. This study involves the innovative ECHO model intervention that addresses barriers to the care of miners by providing a multidisciplinary community of practice approach, which has been well studied in other diseases [17,25-27]. This study is topical and significant because it addresses a critical gap related to the emerging pneumoconiosis epidemic in rural United States. Since no one refused participation, there was likely no potential participation bias in this study. Since the ECHO model has been adopted nationwide and worldwide to care for patients with numerous chronic diseases, infrastructure already exists to rapidly scale the Miners’ Wellness ECHO Program nationwide and worldwide. Other strengths include the detailed contemporaneous interpretation of videos and transcripts of discussions; strict quality control in qualitative analysis; a mixed methods study design; and the simultaneous use of qualitative, quantitative, and subject content experts as coinvestigators.

Although our study focused on the ECHO program, which emphasizes chronic disease management with a strong focus on mentorship and collaborative learning, our findings have broader implications for other digital communities of practice that may be structured differently and operate differently. Examples include Elpha (Women in Tech Network), a community of practice for women in technology; Stack Overflow (Stack Exchange Inc) for programmers and developers; Digital Nomad World for nonprofessional digital nomads [31]; and various leadership communities of practice that emphasize succession planning and leadership skills development [32]. Such unrelated communities of practice can also benefit from our evidence-based findings and recommendations to reach out to silent groups, break down silos, provide facilitator training, and encourage active participation. On the other hand, the ECHO program could learn from other communities of practice on how to reward active participation and knowledge sharing (Stack Overflow) or create asynchronous platforms to supplement synchronous engagement (Digital Nomad World).

There are also limitations to this study. We cannot correlate interprofessional discussion for knowledge transfer to patient outcomes or changes in provider behavior. However, we have previously published a listing of qualitative changes our ECHO participants reported they would make in their practice, obtained as part of a continuing medical education survey requested at the end of each ECHO session [20]. Participants whose group identity was other or unknown were 11.7% (63/539) of the total participant representation in all sessions. When analyzing participant group identity (as shown in Table 4), the other or unknown group was excluded. Although a small sample size raises the possibility of a type I error, individual professionals and teams of professionals trained in the ECHO model can reach a large number of miners, with the potential for creating exponential change. Miners impacted with pneumoconiosis do not participate in the ECHO model. This model characteristic is, however, not a limitation for the following reasons. The model ensures that knowledge is disseminated widely and effectively by focusing on provider education. By reaching out to more miner professionals, the ECHO model indirectly benefits more miners and is more scalable than traditional telemedicine models focusing on individual patients. Case-based learning, where providers present anonymized patient cases to a panel of experts, allows for in-depth discussion and learning without compromising patient confidentiality while maintaining the traditional provider-patient relationship [25,33].

### Conclusions

Our findings provide insight into the mechanics of interprofessional discussion for knowledge transfer in a digital community of practice managing pneumoconiosis and potential recommendations to enhance the same. Our results underscore the capacity of the Project ECHO model to leverage technology and workforce diversity to facilitate interprofessional discussions on the multidisciplinary and complex care of miners and ultimately promote health equity among rural and medically underserved mining communities. Although this approach addresses a critical gap related to the emerging pneumoconiosis epidemic, future research will evaluate whether this translates into improved patient outcomes in mining communities, a priority need in rural United States.

## Supplementary material

10.2196/67999 Multimedia Appendix 1 Example conversations and details of individual ECHO (Extension for Community Health Outcomes) session content.

## Abbreviations

ECHO: Extension for Community Health Outcomes
REDCap: Research Electronic Data Capture
