# Supplementary material for: Interprofessional Discussion for Knowledge Transfer in a Digital “Community of Practice” for Managing Pneumoconiosis: Mixed Methods Study
Source: JMIR Form Res. 2025 Mar 13;9:e67999. doi: 10.2196/67999 (PMC11924965; doi:10.2196/67999)
Supplement: Multimedia Appendix 1 [file formative-v9-e67999-s001.docx]

| Table S1. Three example conversations demonstrating statements of expertise (shown in blue font) and responses of acceptance and eschewal (shown in green and red fonts respectively). |
| --- |
| **Key and definitions** |
| Expertise: Statements of expertise include general utterances of expertise, defining a solution, and answering questions. Anyone who speaks as an expert is an expert.  Accept: Acceptance to statement of expertise, which includes comments like, “Thank you,” “I agree,” or adding to another’s statement of expertise.  Eschew: Eschewal to statement of expertise, which includes comments like, “I disagree,” or providing new “expertise” that contradicts previously stated opinions/information/evidence/statements. Can include partial disagreement/eschewal.  **Note**: Facilitator utterances did not get coded. |
| Case Presentation: *Deposition Dilemmas on Causation and Attribution* Presented by a clinical provider. |
| **Clinical Provider 1:** Do you think long-term smoking causes significant interstitial lung disease like pulmonary fibrosis?    **Clinical Provider 2:** I work in the Interstitial Lung Disease Clinic and it's a well-known risk factor. I mean, tobacco smoking is a very well-known risk factor for UIP or idiopathic pulmonary fibrosis, so yes.  **Respiratory Therapist:** The answer that I get is when I-- for either doctor, would be that yes, smoking can cause fibrosis, but not to this extent.  **Clinical Provider 1:** No. [Clinical Provider 2], because UIP, that's pretty extensive. I never thought that smoking could cause usual interstitial pneumonitis.    **Clinical Provider 2:** So that's what you mentioned about the difference between the medical and the legal understanding of causality, it is a well-known risk factor. But do smokers typically present with pulmonary fibrosis? The answer is no. I mean, majority of them present with emphysema and chronic bronchitis rather than extensive fibrosis. But it's understood that it adds to the susceptibility of that individual to get pulmonary fibrosis. When I see it in medical practice, I mean, I generally do not think of it as a cause, but rather a risk factor. So if there is a legal difference there in the understanding of risk factors and causes, then you probably know more of that as an attorney than I do. |
| Didactic: *Home Pulmonary Rehabilitation – Is it Useful?* Presented by home health professionals. |
| **Clinical Provider:**  There was a recent article that came out in the BMC Pulmonary Medicine journal… And some of their conclusions is kind of supporting what you were suggesting. So one of the things they found was that in community-based pulmonary rehab, they found statistically significant effects addressing patients' mental and psychosocial needs. And something kind of comes to mind - I don't know if it's available to the patients that you treat - is if they are able to use Medicaid, Medicare van type of services to make it to the hospital because it's definitely true if you get everybody in one setting together, you can have a greater impact…    **Facilitator:**  So let me summarize. What you said is that community rehab maybe superior to in-home rehab. And I think the real problem here is that there is no community rehab. So what do you do when you don't have community rehab? …are the two rehabs comparable? It seemed that the community rehab has far greater advantages than in-home rehab.  **Home Health Professional 2:** …I just wanted to make a comment about the home versus the group dynamic. And group dynamic actually, I think, could be not as good as the home and the fact that they are competitive and are trying to out show each other. Men, they don't want to be outdone, but then you're going to get skewed results. You're not going to see what they normally do at home… I did my first visit with a miner this week. And on the depression scale and stuff like that, one of the things I noticed is it asks about sex life, where some older gentlemen might be offended by that, think, "That's none of your business," or something...He states he's depressed all the time but it doesn't really show on my scale because he does try to stay active with his family as much as possible…he misses is his wife. He goes to visit her grave every single day and many times cries, still after three to four years…So maybe if we could ask some questions about missing a spouse or something and how that affects their depression level.  **Facilitator:** Okay. So there are two questions…The first one is that the competitive spirit may actually skew the results in favor of outpatient pulmonary rehab, and, two, that the depression scale does not adequately address the fact that partners and spouse may be dead or missing…  **Home Health Professional 3:** I'd like to answer at least the depression. We had to find a tool that kind of hit kind of the high points of depression. We wanted to see-- I would love to be able to say, "Yes. They answered all the questions about a spouse and kind of localize their depression." However, for what we were using it for, I really couldn't find a tool that can meet that requirement. We needed something broad-based to be able to kind of use it as a measurement tool. So that was the reason why we used the certain tool we do.  **Facilitator:** And, [Home Health Professional 3] if you modify a tool, then you have to validate it, which creates a lot of problems. So for practical purposes, yes, that's a limitation of the tools but that is what you have.    **Home Health Professional 3:** Yes. |
| *Case Presentation: Obesity vs Lung Disease in a Miner*Presented by a clinical provider. |
| **Clinical Provider 1:** And in 2019, the 600-pound gorilla in this discussion is a CT scan of the chest, high resolution may clear up very much the presence of any underlying lung disease. Now, whether that is part of what's standard admission of evidence in assessment of these cases or not, I don't know. Probably should be in 2019, although a lot of times, we look at things like B reads of chest X-rays, which we know are very poor tests to identify interstitial lung disease.  **Facilitator**: Very good. So [Clinical Provider 1] points out that a high-resolution CT scan of the chest may allow us to make a diagnosis of early interstitial lung disease, although we learned in the last presentation that small airways disease can still be missed out even if you did get inspiratory and expiratory views in the high-resolution CT.  **Clinical Provider 1:** Understood, but this isn't a small airways disease case. This is a case of interstitial lung disease.  **Facilitator:** Got it. Okay...I'm just going to summarize [the case]. This is a smoker and a miner who has chronic bronchitis symptoms with dyspnea on exertion, a normal lung exam with morbid obesity, and a restrictive lung disease demonstrated by severely reduced total lung capacity, supportive pre- and post-bronchodilator spirometry, borderline diffusing capacity, an increased alveolar-arterial gradient on the blood gas at rest with a B read that was 0/1 profusion score. And the question that was being asked of us is, "Is this all explained by obesity, or is there any chance that this could be black lung?" And if it is black lung, the things that were pointed out by [Clinical Provider 1] is early interstitial lung disease, pointed out by [Clinical Provider 2] is small airways disease due to coal mine dust exposure, and by [Clinical Provider 3] which is pulmonary hypertension due to whatever causes. So [Attorney], you've seen these kind of cases. What do you do, and what do you advise us to do and what do the coal company normally do?  **Attorney**: …I agree it's a difficult case. With this degree of obesity, I think it's likely that it does contribute to the restrictive impairment, but what I look at when I see a case like this is can the man be entitled to benefits?...we have a negative chest X-ray, so I take it that we cannot prove he has clinical pneumoconiosis… Does he have legal pneumoconiosis? I think one important factor we have to look at is that he does have 22 years of coal mine employment…then with this degree of impairment, he is totally disabled, so he would qualify for the presumption that he does have pneumoconiosis. In this case, it would be legal pneumoconiosis; therefore, the burden would shift to the employer to prove he does not have pneumoconiosis…Obviously, they're going to argue the obesity [and] that there's damage to the hemidiaphragm…Now, what I like to do in these cases is send the gentleman out for a sniff test…It's a fairly simple test, and it's easy to show that he does have good function of his diaphragm…Typically, [employers will also] say, "Well, if you have a restrictive impairment, you're either going to have complicated pneumoconiosis or a high perfusion of simple pneumoconiosis," if it's due to pneumoconiosis, which we don't have here. But I think we have to remember that the regulation defines legal pneumoconiosis as either a obstructive or restrictive impairment, so, by the regulations, we don't have to have a diagnosis of clinical pneumoconiosis. So I think as far as from a medical standpoint diagnosing pneumoconiosis, I agree you're going to have a difficult point, but if the goal is to have him awarded benefits, then I think he does have a reasonable chance of receiving black lung benefits.  **Facilitator**: Very nice. So [Attorney] gives a completely different perspective on this. What he's pointed out was that this person has legal pneumoconiosis, which is chronic bronchitis and undefined restrictive lung disease, and this would qualify him for totally disabling benefits. And the burden of proof shifts to the coal company that he does not have legal pneumoconiosis. That's an incredible, and a very different, perspective. So you would advise us to proceed with filing for benefits, which has been done. But you also said that additional workup should be considered. [Clinical Provider 1] said CT. You said evaluate for neuromuscular disease. Others have talked about an echocardiogram. And [Respiratory Therapist] warned us that the CT may be normal. And if the CT's normal, it still doesn't hurt the patient, does it, [Respiratory Therapist]?  **Respiratory Therapist:** I'd just as soon not have it in there. It may not hurt him, but in my opinion, I'm not going to be the one ordering it. But [Attorney] may feel differently.  **Clinical Provider 1:** Let me just make a little comment about medical, just a diagnosis of pneumoconiosis. First of all, studies have been done looking at interstitial lung disease. High-resolution CT scans are extremely accurate in diagnosing it. A very small percentage of patients who truly have interstitial lung disease will have a normal high-resolution CT scan of the chest. It's a few percent, but very small. The second thing I want to bring up is when you interpret pulmonary function testing - and I have to go after this because I have to see patients - this does not show restrictive lung disease. This is a restrictive defect on the test. It is a complete different statement to say a person has restrictive lung disease compared to pulmonary function testing show a restriction or a restrictive defect on testing.  **Facilitator:** So [Clinical Provider 1], if you have a reduced total lung capacity, would you not say this was restrictive lung disease?  **Clinical Provider 1:** I would not because it's a restrictive defect on the test that lung disease requires other evidence besides this to prove that, when it comes to interstitial lung disease. Just like you cannot take a PFT and say a person has asthma based on PFT as a clinical diagnosis. And you can have an obstructive defect on your exam, but the clinical diagnosis of asthma requires other medical evidence.  **Facilitator:** Thank you, [Clinical Provider 1]. |

**Table S2.** Details of individual ECHO session content during the study timeframe, providing more context to data shown in Tables 1 and 2 and Figure 1 in the manuscript. Examples of conversations demonstrating statements of expertise and responses of acceptance and eschewal are shown in Table S1.

| **Session** | **Didactic topic** | **Case discussion topic** |
| --- | --- | --- |
| 1 | Are the Proposed New Silica Standards Useful? | Is sleep apnea consequential to miners’ lung disease? |
| 2 | ABC’s of ABG | Deposition dilemmas on causation and attribution |
| 3 | Home Pulmonary Rehabilitation – Is it Useful? | What is the benefit of in-home PR in a miner living in a geographically isolated community? |
| 4 | Clearing the air: Consequential conditions in miners | Is dementia consequential to chronic lung disease in miners? |
| 5 | Surface mine jobs and the myth about ‘clean’ exposure | Posttraumatic pleural changes as legal pneumoconiosis? |
| 6 | Controversies in the management of Chronic Beryllium Disease | Steroids and False Negative Beryllium Tests |
| 7 | Rheumatoid arthritis and mining exposure - causation and confounding dilemmas | When is scleroderma secondary to silica exposure? |
| 8 | Common mistakes clinical providers make in assessing respiratory impairment | Can non-obstructive chronic bronchitis cause total disability? |
| 9 | Cor Pulmonale in miners | When can one reasonably make a diagnosis of cor pulmonale? |
| 10 | Labor laws and home care for miners | Is the miner’s home health caregiver responsible for paying income tax? |
| 11 | Why sleep apnea matters in miners? | How does one determine qualifying PFT values for DOL compensation for miners over age of 71 years, which is where the age column in the DOL tables stop? |
| 12 | Identifying small airways disease in miners | Is isolated diffusion impairment consistent with Coal Mine Dust Lung Disease? |
| 13 | How to ace your spirometry test? | What are the main challenges DOL patients face when trying to get oxygen? |
| 14 | Histoplasmosis & Coccidiomycosis – Confounders in dust-related lung diseases | Obesity vs. lung disease in a miner |
| 15 | Determining disability in federal black lung | Is a miner with a disabling ABG at rest but not disabling ABG on exercise have disabling pneumoconiosis? |
| 16 | MAC and the miners' lungs | When totally disabling spirometry is neither acceptable nor repeatable. |
| 17 | Differentiating respiratory from cardiovascular limitation to exercise | Does vaping impact lung function findings and diagnosis in a miner? |
| 18 | Lessons learned from the regional variability in the progressive massive fibrosis epidemic | Rounded atelectasis as complicated pneumoconiosis in a coal miner? |
| 19 | Why pilots crash? Making medical opinions by physicians fly! | Can six minute walk test indicate impairment without qualifying resting tests? |
| 20 | Legal pneumoconiosis: Why does it confuse physicians? | Newer therapies for the frequent exacerbator phenotype of (occupational) COPD |
| 21 | Why the variability in B read interpretation of chest radiographs? | Can profusion scores on chest X-ray decrease over time? |
| 22 | Six minute walk test and assessing impairment | Can you estimate a miner’s peak oxygen consumption by six minute walk distance to rate impairment? |
| 23 | HIPAA compliance and care of miners | What are appropriate tests for an oxygen DME request? |
